# Real Life Evolution of Surgical Approaches in the Management of Endometrial Cancer in Poland

**DOI:** 10.3390/cancers17162626

**Published:** 2025-08-11

**Authors:** Agnieszka Rychlik, Tomasz Kluz, Grzegorz Szewczyk, Pluvio J. Coronado, Tomasz Łatkiewicz, Rafał Tarkowski, Anna Woińska-Przekwas, Krzysztof Nowosielski, Kaja Skowronek, Rafał Stojko, Michał Skuza, Marcin Misiek, Krzysztof Jabłoński, Paweł Sadłecki, Marta Ciosek, Katarzyna Pasicz, Anna Bogaczyk, Mariusz Bidziński

**Affiliations:** 1 Department of Gynecologic Oncology, National Research Institute of Oncology, 02-781 Warsaw, Polandbidzinski.m@gmail.com (M.B.); 2 Department of Gynecologic Oncology, Fryderyk Chopin University Hospital, 35-055 Rzeszow, Poland; jtkluz@interia.pl (T.K.); annabogaczyk@interia.pl (A.B.); 3 Faculty of Medicine, University of Rzeszów, 35-310 Rzeszow, Poland; 4 Department of Obstetrics, Gynecology and Gynecological Oncology, Mazovian Provincial Hospital, 08-110 Siedlce, Poland; awoinska@szpital.siedlce.pl; 5 1st Department of Obstetrics and Gynecology, Centre for Medical Postgraduate Education, 01-813 Warsaw, Poland; 6 Women’s Health Institute, San Carlos Clinic Hospital, IdISSC, Faculty of Medicine, Complutense University, 28040 Madrid, Spain; plujcoro@ucm.es; 7 The First Department of Gynecologic Oncology and Gynecology, Medical University of Lublin, 20-059 Lublin, Poland; tomek.latkiewicz@gmail.com (T.Ł.); rafaltar@yahoo.com (R.T.); 8 Department of Gynecology, Obstetrics and Gynecological Oncology, University Clinical Center, Medical University of Silesia, 40-055 Katowice, Poland; knowosielski@sum.edu.pl; 9 Department of Obstetrics and Gynecology with the Subdivision of Oncological Gynecology, Brothers Hospitallers of Saint John of God Hospital in Katowice, 40-635 Katowice, Poland; skowronek.kaja@gmail.com (K.S.); rafal@czkstojko.pl (R.S.); 10 Department of Gynecologic Oncology, Holly Cross Cancer Center, 25-734 Kielce, Poland; michalskuza89@gmail.com (M.S.); mmisiek@me.com (M.M.); 11 Department of Gynecology and Gynecologic Oncology, Municipal Polyclinical Hospital in Olsztyn, 10-045 Olsztyn, Poland; jabkrys@op.pl; 12 Department of Gynecologic Oncology, Municipal Polyclinical Hospital in Grudziadz, 86-300 Grudziądz, Poland; 13 Faculty of Medicine, University of Science and Technology, 85-796 Bydgoszcz, Poland; 14 Department of Gynecology, Holy Family Hospital, 02-544 Warsaw, Poland; marta.e.ciosek@gmail.com; 15 Medical Physics Department, National Research Institute of Oncology, 02-781 Warsaw, Poland; katarzyna.pasicz@nio.gov.pl

**Keywords:** endometrial cancer, surgery, minimally invasive surgery

## Abstract

**Objective:** The primary objective of this study was to evaluate the evolution of surgical approaches in the management of endometrial cancer in Polish tertiary referral hospitals, comparing the use of minimally invasive surgery (MIS) and laparotomy in 2023 versus 2013. **Methods:** This retrospective observational study analyzed data from tertiary referral centers in Poland. All surgeries performed for apparently early-stage endometrial cancer in 2013 and 2023 were included. **Results:** A total of 1062 patients were analyzed, with 417 undergoing operations in 2013 and 640 in 2023. In 2013, 92.6% (386/417) of patients underwent laparotomy. By 2023, 80.1% (513/640) of patients were treated using minimally invasive approaches, including laparoscopy (56.2%, 362/640), robotic-assisted laparoscopy (21.7%, 139/640), and vaginal surgery (1.9%, 12/640). No conversions to laparotomy were recorded in 2013. In 2023, 22 conversions occurred—21 in the laparoscopy group (5.8%, 21/362) and one in the vaginal surgery group (8.3%, 1/12). No conversions were reported in the robotic-assisted group. Intraoperative complications were observed in 2.2% (8/362) of laparoscopic cases, and postoperative complications in 4.4% (16/362). In the robotic-assisted group, one intraoperative complication (0.7%) was reported, with no postoperative complications. **Conclusions:** Over the past decade, there has been a significant shift in the surgical management of endometrial cancer in Poland, with a growing preference for minimally invasive surgery (MIS). The rate of conversion from MIS to laparotomy remains below 6%. Robotic-assisted laparoscopic surgery may offer additional benefits, particularly for obese patients.

## Simple Summary

The study analyzes a representative group of patients treated for endometrial cancer in the Polish population. It illustrates the radical changes in the surgical treatment of this cancer that have occurred over the past 10 years, reflecting the transition towards minimally invasive surgery and the resulting reduction in perioperative complications. The study also highlights the issue of conversion to laparotomy, its reasons, and the potential benefits of robotic-assisted laparoscopic surgery in this regard. In the presented study, significantly lower conversion rates and postoperative complications were observed in the group of patients operated by robotic assisted laparoscopic surgery when compared to conventional laparoscopy. These hypotheses-generating conclusions should be verified by prospective studies.

## 1. Introduction

Endometrial cancer (EC) is the most prevalent gynecological malignancy in developed countries, with a rising incidence in Poland. In 2022, 5995 cases of EC were registered [1]. Since the publication of the LAP2, LACE, and the Dutch randomized trial [2,3,4], minimally invasive surgery (MIS) has become the gold standard for the surgical management of EC. MIS has been shown to be oncologically safe and superior in terms of the short-term outcomes, including fewer complications and shorter hospital stays. It is endorsed as the standard approach in international guidelines, including those of the European Society of Gynecologic Oncology (ESGO) and the European Society of Medical Oncology (ESMO) [5,6], and the rate of MIS is a major parameter of the ESGO quality indicators in the management of EC [7]. The three major studies that confirmed the feasibility and safety of laparoscopy in endometrial cancer were conducted over a decade ago. Nevertheless, their findings on conversion rates from laparoscopy to laparotomy, morbidity, mortality, and hospital stay duration remain key reference points. In the LAP2 study published in 2009, 25.8% of patients randomized to laparoscopy required conversion to open laparotomy, with poor exposure cited as the primary reason. In the Dutch trial, conversion occurred in 10.8% (20 of 185) of laparoscopic procedures. A later analysis of the LACE trial reported a 6% conversion rate, mostly due to anatomical factors. ESGO has established a conversion rate of less than 10% as a quality benchmark for endometrial cancer surgery. Patients who undergo conversion are more likely to experience surgical site infections, require reoperations or blood transfusions, and have longer hospital stays. Despite strong evidence supporting the benefits of MIS, it took more than two decades for laparoscopy to become a standard of care in many countries, including Poland. According to a survey conducted by the National Consultant for Obstetrics and Gynecology, only 9% of hysterectomies in Poland were performed using MIS techniques in 2013 [8].

In the context of endometrial cancer surgery, despite robust scientific evidence, MIS was rarely performed in Poland in 2013. However, its adoption has increased significantly in the years since. The primary objective of this study was to assess the evolution of surgical approaches and the rate of conversion from laparoscopy to laparotomy in endometrial cancer procedures performed at Polish tertiary referral hospitals between 2013 and 2023. As secondary objectives, this study analyzed factors associated with conversion from MIS to laparotomy, including patient age, body mass index (BMI), surgical history, FIGO stage, and type of surgical approach. Intraoperative and postoperative complication rates were also assessed. Additionally, a comparison was made between conventional laparoscopy (LPS) and robotic-assisted laparoscopic surgery (RALS) in terms of conversion rates and postoperative complications.

## 2. Materials and Methods

This observational retrospective study is based on data collected from voluntary tertiary centers in Poland. It includes all patients diagnosed with apparent FIGO stage I or II endometrial cancer (EC) who underwent surgery in 2013 and 2023 at these centers. All patients received treatment covered by public health insurance.

Surgical procedures included hysterectomy with or without sentinel lymph node biopsy, or hysterectomy with pelvic lymph node dissection, with or without para-aortic lymphadenectomy.

Conventional laparoscopic procedures were performed by either general gynecologists or gynecologic oncologists. All robot-assisted laparoscopic surgeries (RALS) were conducted by gynecologic oncologists using either the Da Vinci Xi System (available in one center) or the Surgical Versius Robotic System (available in two centers). Policies regarding the use of uterine manipulators varied between centers and were not considered in this study.

Patients with advanced-stage EC, as determined during the preoperative assessment—including serosal, parametrial, nodal, vaginal, or adnexal involvement (FIGO 2009 stage III); metastatic disease (FIGO 2009 stage IV); uterine sarcomas; or those who received chemotherapy or radiotherapy as primary treatment—were excluded from the study.

Data collected included the patient age, body mass index (BMI), preoperative and postoperative FIGO 2009 stages, history of previous laparotomy, and intraoperative and postoperative complications.

### Statistics

Qualitative variables are presented as frequencies and percentages. Quantitative variables are summarized using the mean and standard deviation (SD). Associations between qualitative variables were assessed using the Chi-squared test or Fisher’s exact test, as appropriate. Odds ratios (ORs) with 95% confidence intervals (95% CI) were calculated. To analyze relationships between study groups and continuous variables with a parametric distribution, Student’s *t*-test or an analysis of variance (ANOVA) was used. For non-parametric distributions, the Mann–Whitney U test or Kruskal–Wallis test was applied.

## 3. Results

A total of 1027 patients from ten tertiary centers in Poland were included in the analysis. Of these, 640 patients underwent surgery in 2023 and 417 in 2013. The mean ages were 63.7 ± 10.5 and 65.2 ± 10.3 in 2013 and 2023, respectively. The mean BMI in 2023 was 31.2 ± 7.1, with 302 out of 640 patients (51.1%) having a BMI greater than 30. In 2013, the mean BMI was also 31.2 ± 7.1, with 187 out of 417 patients (44.8%) classified as obese (BMI > 30).

Intraoperative complications were reported in 15 patients in 2013 (15/417, 3.6%) and in 25 patients in 2023 ((25/640, 3.9%); OR: 1.01 (95% CI 0.68–1.49)). Postoperative complications occurred in 49 patients in 2013 (49/417, 11.7%) and in 31 patients in 2023 ((31/640, 4.8%); OR: 0.60 (95% CI 0.50–0.72)).

General characteristics of patients from both cohorts are presented in Table 1.

**Figure 1:**
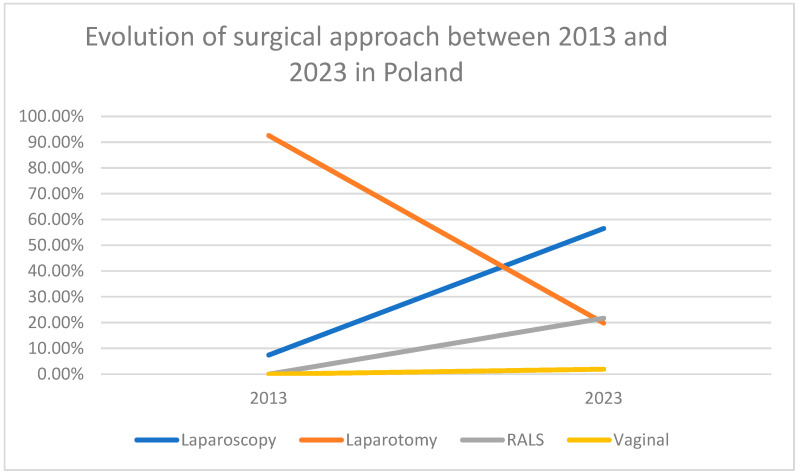
Evolution of surgical approaches between 2013 and 2023 in Poland.

In 2013, 92.6% of patients (386/417) underwent surgery via laparotomy, while 7.4% (31/417) were treated using LPS. In 2013, only one high-volume center performed a notable number of laparoscopic procedures, in 20 out of 68 cases (29.4%). Two other centers performed 5 and 4 laparoscopic surgeries, respectively out of 72 (6.9%) and 64 (6%) procedures, and one center performed 2 out of 65 (3%). The remaining six centers did not perform any laparoscopic procedures during that year. By 2023, the distribution of surgical approaches had shifted considerably, whereby 56.5% (362/640) of patients were operated using LPS, 21.7% (139/640) using RALS, and 1.9% (12/640) via the vaginal route. In total, MIS accounted for 80.1% (513/640) of all procedures, whereas laparotomy was performed in 19.8% of cases (127/640). The centers that performed laparoscopic procedures in 2013 respectively carried out the following number of such procedures in 2023: 91 of 95 (95.7%), 129 of 142 (90.8%), 48 of 48 (100%), and 65 of 82 (79.2%). A detailed overview of surgical approaches is provided in Table 2 and illustrated in Figure 1.

Postoperative complications were statistically more frequent among patients operated on in 2013, with 52 cases compared to 31 in 2023 (*p* < 0.001). Infection-related complications were also more prevalent in 2013, occurring in 36 patients (8.6%) versus 12 patients (1.9%) in 2023. A summary and comparison of the postoperative complications is presented in Table 3.

There were no conversions to laparotomy among the 31 patients operated on laparoscopically in 2013. In contrast, in 2023, 21 conversions occurred within the LPS group (21/362, 5.8%) (Table 4). One conversion was recorded in the vaginal surgery group (1/12, 8.3%), while no conversions were observed in the RALS group (0/139).

When analyzing the reasons for conversion, nine cases were attributed to adhesions and five to suboptimal exposition. One conversion was due to peritoneal spread not identified during the preoperative workup, and another was related to anesthetic concerns. Additionally, nine conversions resulted from intraoperative complications—seven due to bleeding and two due to urinary tract injury. A detailed summary of the conversion reasons is presented in Table 5.

The characteristics of the conversion group are presented in Table 6. The mean age in this group was 64.7± years, and the mean BMI was 32.6 ± 6.1. A total of 81.8% of patients (18/22) had a BMI over 30. Nine patients (40.9%) had a history of previous laparotomy. Intraoperative complications occurred in five cases (5/22, 22.7%), and two postoperative complications were recorded. The five intraoperative complications included two cases of excessive blood loss and three urinary tract injuries. Among the three postoperative complications, one case each of bowel injury, infection, and postoperative bleeding was observed (Table 6).

When comparing the RALS and LPS groups from 2023, similar mean ages were observed, with 63.9 (SD 9.1) years in the RALS group and 65.7 (SD 10.5) years in the LPS group. The mean BMI values were also comparable, at 32.1 (SD 6.1) in the RALS group and 31.5 (SD 6.8) in the LPS group.

A history of previous laparotomy was reported in 26.8% of patients in the LPS group (97/362) and in 22.3% of patients in the RALS group (31/139), and this was a significant reason for the conversion in the LPS group (OR intraoperative complications occurred in 2.4% of LPS procedures (9/362) and in 0.7% of RALS procedures (1/139)). Postoperative complications were diagnosed in 5.2% of patients in the LPS group (19/362), while no postoperative complications were reported in the RALS group. A detailed comparison of the RALS and LPS groups is presented in Table 7.

## 4. Discussion

### 4.1. Principal Findings—Shift to Minimally Invasive Surgery

This study confirms a significant shift from open surgery to minimally invasive surgery in the treatment of endometrial cancer in Poland between 2013 and 2023. This remarkable transition is accompanied by a reduction in postoperative complications and aligns with previously published findings [9,10].

### 4.2. Rate of Conversions

The conversion rate in 2023 was estimated at 5.8% in the LPS group, with no conversions reported in the RALS group. The primary hypothesis of this study could not be confirmed, likely due to the low rate of MIS and potential patient selection bias in 2013.

Notably, the low conversion rate observed in the participating centers is consistent with ESGO quality indicators, which recommend that conversions to laparotomy due to intraoperative findings or complications remain below 10% [7].

In this study, the most common reason for conversion was adhesions, consistent with findings reported in previous studies [11,12]. Other authors have identified a large uterus as the leading cause of conversion (27.0%), followed by extensive adhesions (24.3%) and surgical complications (18.9%) [13].

### 4.3. Impact of RALS

In our study, no conversions to laparotomy and no postoperative complications were observed in the RALS group. One intraoperative complication was reported in this group, with a statistically significant difference when compared to conventional laparoscopy.

In contrast, a Cochrane review pooling data reported no significant difference in conversion rates between the two approaches (RR 1.17, 95% CI 0.24 to 5.77) [14]. Conflicting results were presented by Mäenpää et al. [15], who in a small prospective study comparing RALS and LPS in endometrial cancer reported no conversions in the RALS group. However, the conversions observed in the LPS group were unrelated to the surgical approach, limiting the validity of the study’s conclusions.

This specific question is currently being addressed in the ongoing prospective randomized phase III RObese trial [16]. A recently published meta-analysis suggests superior outcomes for RALS in achieving complete lymph node staging in severely obese patients with endometrial cancer, while LPS appears to be associated with fewer postoperative complications [17].

The introduction of RALS has expanded the number of surgeons capable of performing MIS for endometrial cancer. However, no clinical benefit of RALS in terms of survival, when compared with conventional laparoscopy (LPS), has been demonstrated in either retrospective or prospective studies [18,19].

Moreover, prospective evidence has not demonstrated that RALS is superior to LPS with respect to the incidence of severe perioperative morbidity in patients with gynecologic cancer, and it is associated with longer operating times [20].

Nonetheless, several retrospective studies have highlighted potential benefits of RALS [21,22,23]. For instance, Coronado et al. reported fewer postoperative complications, shorter hospital stays, and reduced procedural complexity, which may particularly benefit frail populations such as elderly patients [21].

Various theories have been proposed regarding the added value of robotic surgery. One such theory highlights the advantage of the robot’s wristed instruments, which enhance the exposure and surgical technique. The EndoWrist^®^ technology enables surgical maneuvers comparable to those used in open surgery, allowing less experienced surgeons—particularly those with limited laparoscopic skills—to perform complex tasks such as intracorporeal suturing and knot-tying. Enhanced visualization, the additional robotic arm, and improved dexterity in confined spaces, such as those encountered in obese patients, offer a clear advantage of RALS over LPS [21].

RALS is also associated with a relatively shorter learning curve. The number of cases required to achieve proficiency in RALS for endometrial cancer surgery is estimated at approximately 20 [24]. This has been attributed by some investigators to prior experience in advanced laparoscopic procedures, which may facilitate earlier proficiency in robotic surgery [24,25,26].

### 4.4. Impact of Obesity

It has been suggested that RALS may offer advantages in the treatment of obese women with endometrial cancer, including reduced blood loss, lower conversion rates to laparotomy, and decreased procedural complexity [27]. However, these findings were not confirmed in a comprehensive systematic review and meta-analysis. However, the authors of that review did note that robotic hysterectomy may reduce conversions related to positional intolerance in patients with morbid obesity [28]. In this patient population, the most common reason for conversion—both in laparoscopic and robotic hysterectomy—remains inadequate exposure due to adhesions or visceral adiposity.

In our study, despite the higher mean BMI in the RALS group, no conversions were observed among patients operated using this technique. Notably, the median BMI in the conversion group was higher than in the overall study population, supporting the association between elevated BMI and increased odds of conversion.

One possible explanation is that RALS can be performed with reduced intra-peritoneal pressure, owing to the fixed mechanical arms of the robotic system that support the weight of the abdominal wall. This feature may contribute to a lower rate of complications, including those classified in our study as anesthetic-related conversions.

A low-pressure surgical technique has also been described for conventional laparoscopy in morbidly obese patients with endometrial cancer. A group from Milan developed a sophisticated subcutaneous abdominal wall retraction device, the Laparo–Tenser device (Lucini Surgical Concept srl, Milan, Italy), which enables low-pressure laparoscopy. However, its high cost has limited its widespread adoption [29].

### 4.5. Strength and Weaknesses of the Study

The strength of this study lies in the large number of patients included, representing approximately 10% of all endometrial cancer cases treated in Poland in 2013 and 2023. The geographical distribution of participating centers spans six different regions of the country. Patients were recruited from comprehensive cancer care centers, a university hospital, and two community hospitals. All patients treated in each center during the study period were included, providing an illustrative and representative sample.

However, the retrospective nature of the study constitutes a clear limitation. Some patients from the 2013 cohort lack post-operative staging due to incomplete records. At that time, hospital information systems were not widely used, and data were often unavailable in both electronic and paper archives. It should also be noted that robotic surgery systems were available in only three out of the ten participating centers, and the level of surgical proficiency was not assessed. These unmeasured confounding variables represent major limitations and may have influenced the observed impact of RALS. Therefore, the results should be interpreted with these limitations in mind.

## 5. Conclusions

Minimally invasive surgery has been widely adopted in Poland over the past decade, and the benefits of this transition are reflected in improved perioperative outcomes. Conversion rates are no longer a major concern in MIS for endometrial cancer. However, the potential advantages of RALS in this context require confirmation through prospective studies.

## Figures and Tables

**Table 1:** General characteristics of the whole study group (NA—data not available).

		2013 N = 417	2023 N = 640	*p* Value
Age (mean ± SD)		63.7 ± 10.5	65.2 ± 10.3	0.027
BMI (mean ± SD)		31.4 ± 7.1	31.2 ± 7.1	0.712
Previous laparotomy		109 (26.1%)	192 (30%)	0.007
FIGO 2009 preoperative stage				0.013
	IA	221 (53%)	370 (57.8%)	0.139
	IB	110 (26.4%)	192 (30%)	0.229
	II	20 (4.8%)	67 (10.5%)	0.002
	NA	66 (15.8%)	11 (1.7%)	<0.001
FIGO 2009 postoperative stage				0.608
	IA	135 (32.3%)	329 (51.4%)	<0.001
	IB	64 (15.3%)	150 (23.4%)	0.002
	II	46 (11.1%)	85 (13.3%)	0.322
	IIIA	6 (1.4%)	10 (1.6%)	0.999
	IIIB	9 (2.2%)	18 (2.8%)	0.646
	IIIC	20 (4.8%)	44 (6.9%)	0.210
	IV	2 (0.5%)	4 (0.6%)	0.999
	NA	135 (32.4%)	0 (0%)	<0.001
Intraoperative complications		16 (3.8%)	25 (3.9%)	0.990
Postoperative complications		52 (12.5%)	31 (4.8%)	<0.001

**Table 2:** Surgical approaches in 2013 and 2023.

SURGICAL APPROACH	2013	2023
Laparoscopy	31 (7.4%)	362 (56.5%)
Robot-assisted surgery	-	139 (21.7%)
Laparotomy	386 (92.6%)	127 (19.8%)
Vaginal	-	12 (1.9%)
**Total**	**417**	**640**

**Table 3:** Postoperative complications in 2013 and 2023. Bleeding was defined as clinically significant blood loss that necessitated reoperation.

POSTOPERATIVE COMPLICATIONS	2013 N (%)	2023 N (%)	*p* Value	OR (95% CI)
Bleeding	7 (1.7%)	4 (0.6%)	0.367	1.8 (0.5–5.9)
Urinary tract injury	4 (1%)	1 (0.2%)	0.179	4.0 (0.4–35.8)
Bowel injury	1 (0.2%)	3 (0.5%)	0.317	0.3 (0.03–3.2)
Infections	36 (8.6%)	12 (1.9%)	<0.001	3.0 (1.6–5.7)
Other	4 (1%)	11 (1.7%)	0.071	0.4 (0.1–1.1)
**Total**	**52 (12.5%)**	**31** **(4.8%)**	**<0.001**	**0.60** **(0.50–0.72)**

**Table 4:** Conversion according to surgical approach.

CONVERSIONS ACCORDING TO SURGICAL APPROACH in 2023 N = 22		
Total MIS	22/513	4.3%
Laparoscopy	21/362	5.8%
Vaginal	1/12	8.3%
Robot-assisted surgery	0/139	0%

**Table 5:** Reasons for conversion.

REASON OF CONVERSION		
Bleeding	7	28.0 %
Peritoneal spread	1	4.0 %
Urinary/bowel complications	2	8.0 %
Anesthesiological reason	1	4.0 %
Suboptimal exposition	5	20.0 %
Adhesions	9	36.0 %
**Total**	**22**	

**Table 6:** Characteristics of conversion group.

CONVERSION GROUP (N = 22)		
Age (mean ± SD)	66.5 ± 10.9	
BMI (mean ± SD)	32.6 ± 6.1	
Previous laparotomy	9 (40.9%)	
FIGO 2009 preoperative stage		
	IA	13 (59.1%)
	IB	7 (31.9%)
	II	2 (9%)
FIGO 2009 postoperative stage		
	IA	9 (40.9%)
	IB	6 (27.2%)
	II	4 (18.2%)
	IIIA	1 (4.5%)
	IIIB	0
	IIIC	2 (9.1%)
Intraoperative complications	5 (22.7%)	
Postoperative complications	2 (9.1%)	

**Table 7:** Comparison of RALS and LPS group (2023).

	RALS (n = 139)	LPS (n = 362)	*p* Value	OR (95%CI)
Age (mean ± SD)	63.9 ± 9.1	65.7 ± 10.5	0.733	-
BMI (mean ± SD)	32.1 ± 6.1	31.5 ± 6.8	0.329	-
Conversions	0 (0%)	21 (5.8%)	0.016	-
Previous laparotomy	31 (22.3%)	97 (26.7%)	<0.001	15.6 (2.9–83.4)
Intraoperative complications	1 (0.7%)	9 (2.4%)	0.693	0.5 (0.1–2.9)
Postoperative complications	0 (0%)	19 (5.2%)	0.026	-

## Data Availability

The data presented in this study are available on request from the corresponding author. The data are not publicly available because they contain information that could compromise the privacy of patients.

## Abbreviations

The following abbreviations are used in this manuscript:

EC	Endometrial cancer
RALS	Robotic-assisted laparoscopic surgery
LPS	Conventional laparoscopy
MIS	Minimally invasive surgery
ESGO	European Society of Gynecologic Oncology
ESMO	European Society of Medical Oncology
FIGO	International Federation of Gynecology and Obstetrics
BMI	Body mass index
OR	Odds ratio
SD	Standard deviation
